# The IUPHAR/BPS Guide to PHARMACOLOGY in 2016: towards curated quantitative interactions between 1300 protein targets and 6000 ligands

**DOI:** 10.1093/nar/gkv1037

**Published:** 2015-10-12

**Authors:** Christopher Southan, Joanna L. Sharman, Helen E. Benson, Elena Faccenda, Adam J. Pawson, Stephen P. H. Alexander, O. Peter Buneman, Anthony P. Davenport, John C. McGrath, John A. Peters, Michael Spedding, William A. Catterall, Doriano Fabbro, Jamie A. Davies, NC-IUPHAR

**Affiliations:** 1 Centre for Integrative Physiology, University of Edinburgh, Edinburgh, EH8 9XD, UK; 2 School of Biomedical Sciences, University of Nottingham Medical School, Nottingham, NG7 2UH, UK; 3 Laboratory for Foundations of Computer Science, School of Informatics, University of Edinburgh, Edinburgh, EH8 9LE, UK; 4 Clinical Pharmacology Unit, University of Cambridge, Cambridge, CB2 0QQ, UK; 5 School of Life Sciences, University of Glasgow, Glasgow, G12 8QQ, UK; 6 Neuroscience Division, Medical Education Institute, Ninewells Hospital and Medical School, University of Dundee, Dundee, DD1 9SY, UK; 7 Spedding Research Solutions SARL, Le Vésinet 78110, France; 8 Department of Pharmacology, University of Washington, Seattle, WA 98195-7280, USA; 9 PIQUR Therapeutics, Basel 4057, Switzerland

## Abstract

The IUPHAR/BPS Guide to PHARMACOLOGY (GtoPdb, http://www.guidetopharmacology.org) provides expert-curated molecular interactions between successful and potential drugs and their targets in the human genome. Developed by the International Union of Basic and Clinical Pharmacology (IUPHAR) and the British Pharmacological Society (BPS), this resource, and its earlier incarnation as IUPHAR-DB, is described in our 2014 publication. This update incorporates changes over the intervening seven database releases. The unique model of content capture is based on established and new target class subcommittees collaborating with in-house curators. Most information comes from journal articles, but we now also index kinase cross-screening panels. Targets are specified by UniProtKB IDs. Small molecules are defined by PubChem Compound Identifiers (CIDs); ligand capture also includes peptides and clinical antibodies. We have extended the capture of ligands and targets linked *via* published quantitative binding data (e.g. K_i_, IC_50_ or K_d_). The resulting pharmacological relationship network now defines a data-supported druggable genome encompassing 7% of human proteins. The database also provides an expanded substrate for the biennially published compendium, the *Concise Guide to PHARMACOLOGY*. This article covers content increase, entity analysis, revised curation strategies, new website features and expanded download options.

## INTRODUCTION

As demonstrated by this journal special issue, open databases have become indispensable for pharmacology, drug discovery, metabolism and chemical biology, and are increasingly important across other biomedical domains. The amount of structural information now freely available is immensely useful to researchers, but navigating the resources is becoming problematic for database users (1). UniChem and PubChem now exceed 90 and 60 million entries respectively, with nearly 14 million structures added in 2014 alone (2,3). Of these, however, only 0.4% have been tested experimentally. Thus, while just over 2 million of the current PubChem compounds have BioAssay results (with ≈50% tagged as active) (4), the increase in submitted structures is accelerating way beyond the community capacity to generate bioactivity measurements, extract them manually from papers and patents, crowd-source representations for structural correctness, or to curate synonym mappings. This cheminformatics problem is analogous to the situation in bioinformatics, where the gap between the generation of new protein sequences and the experimental assignment of at least some level of biological function is inexorably widening. For example, while UniProtKB/TrEMBL has mushroomed to nearly 50 million entries, only just over 0.5 million entries have supporting evidence for the UniProtKB/Swiss-Prot level of expert annotation (5). While the analogy should not be taken too far, the IUPHAR/BPS Guide to PHARMACOLOGY (GtoPdb, http://www.guidetopharmacology.org; (6)) has some conceptual overlap with Swiss-Prot in that we also seek to maximise the level of data support within our ‘small data’ resource, to underpin the exploitation of ‘big data’. We thus continue to focus our curatorial capacity on a high-quality, annotated subset of human targets with quantitative ligand relationships. These are selected as being the most relevant to contemporary pharmacology and future drug discovery. From its origins in 2011, GtoPdb has become recognized for the following:

- Providing an authoritative and web-browsable synopsis of drug targets and drugs (approved, clinical or research);
- Being an accurate and continually expanding source of information for molecular mechanisms of action (MMOA) of pharmacological agents;
- Facilitating selection of appropriate selective compounds for *in vitro* and *in vivo* experimentation;
- Providing a hierarchical organization of receptors, channels, transporters, enzymes and other drug targets according to their molecular relationships and physiological functions;
- Incorporating nomenclature recommendations from the International Union of Basic and Clinical Pharmacology (IUPHAR) Committee on Receptor Nomenclature and Drug Classification (NC-IUPHAR);
- Utilising a network of NC-IUPHAR subcommittees, comprising over 600 domain experts, to guide ligand and target annotation;
- Inclusion of reciprocal links to key genomic, protein and small molecule resources;
- Monitoring the de-orphanization of molecular targets, particularly receptors;
- Disseminating NC-IUPHAR-derived standards and terminology in quantitative pharmacology;
- Offering advanced query and data mining;
- Providing a variety of downloadable data sets and format options;
- As the source for the biennially published *Concise Guide to PHARMACOLOGY* compendium;
- Being an educational resource for researchers, students and the public.

The sections below will expand on these aspects, focusing on changes since our 2014 publication (6).

## CONTENT EXPANSION

### Targets

Our generic use of the term ‘target’ refers to a record in the database that has been resolved to a UniProtKB/Swiss-Prot ID as our primary identifier. Reasons for this choice include (i) the Swiss-Prot canonical philosophy of protein annotation, (ii) species specificity and (iii) global reciprocal cross-referencing. Notwithstanding, target records also include RefSeq protein IDs and genomic IDs from Entrez Gene, HGNC and Ensembl. Because NC-IUPHAR oversees the nomenclature of (particularly) receptors and channels, these human protein classes are complete in GtoPdb (with the exception of the olfactory and opsin-type GPCRs). The G protein-coupled receptors (GPCRs), ion channels and nuclear hormone receptors (NHRs) were present in the earliest database versions, regardless of the level of molecular pharmacology that could be assigned to them at that time, although they were obviously chosen because they were drug-target rich. By 2012, the catalytic receptors and transporters had been added. At the end of 2012 we received a Biomedical Resources Grant from the UK Wellcome Trust with the objective of capturing the likely targets of future medicines (i.e. to cover the data-supported druggable genome). We consequently embarked on a major expansion of protein capture, of which enzymes formed the largest part. The current category counts are shown in Table 1 (note that statistics of all content types specified throughout this paper refer to our database release 2015.2 from August 2015).

**Table 1.** Target class content

Targets	UniProt ID count
7TM receptors*	395
Nuclear hormone receptors	48
Catalytic receptors	239
Ligand-gated ion channels	84
Voltage-gated ion channels	141
Other ion channels	47
Enzymes (all)	1164
Transporters	508
Kinases	539
Proteases	240
Other proteins	135
Total number of targets	2761

*Not all our 7TM receptor records are unequivocally assigned as GPCRs, but for convenience we refer to these generally as GPCRs in the text.

The total number of targets in Table 1 represents 14% of the current Swiss-Prot human protein count of 20,204; although not all our entries are yet mapped to ligands. While the database is centred on human proteins, information from mouse and rat are also presented because rodent binding data are the most common type encountered in papers, either in addition to or instead of, human data. We thus currently have 6929 human proteins and rat and mouse orthologues (i.e. 84% of a maximum projected three-species count). The 16% shortfall is because either, some do not yet have Swiss-Prot IDs (i.e. are TrEMBL only) or, our curation indicates the orthology relationships are more complex than the 1:1 case.

**Figure 1.**
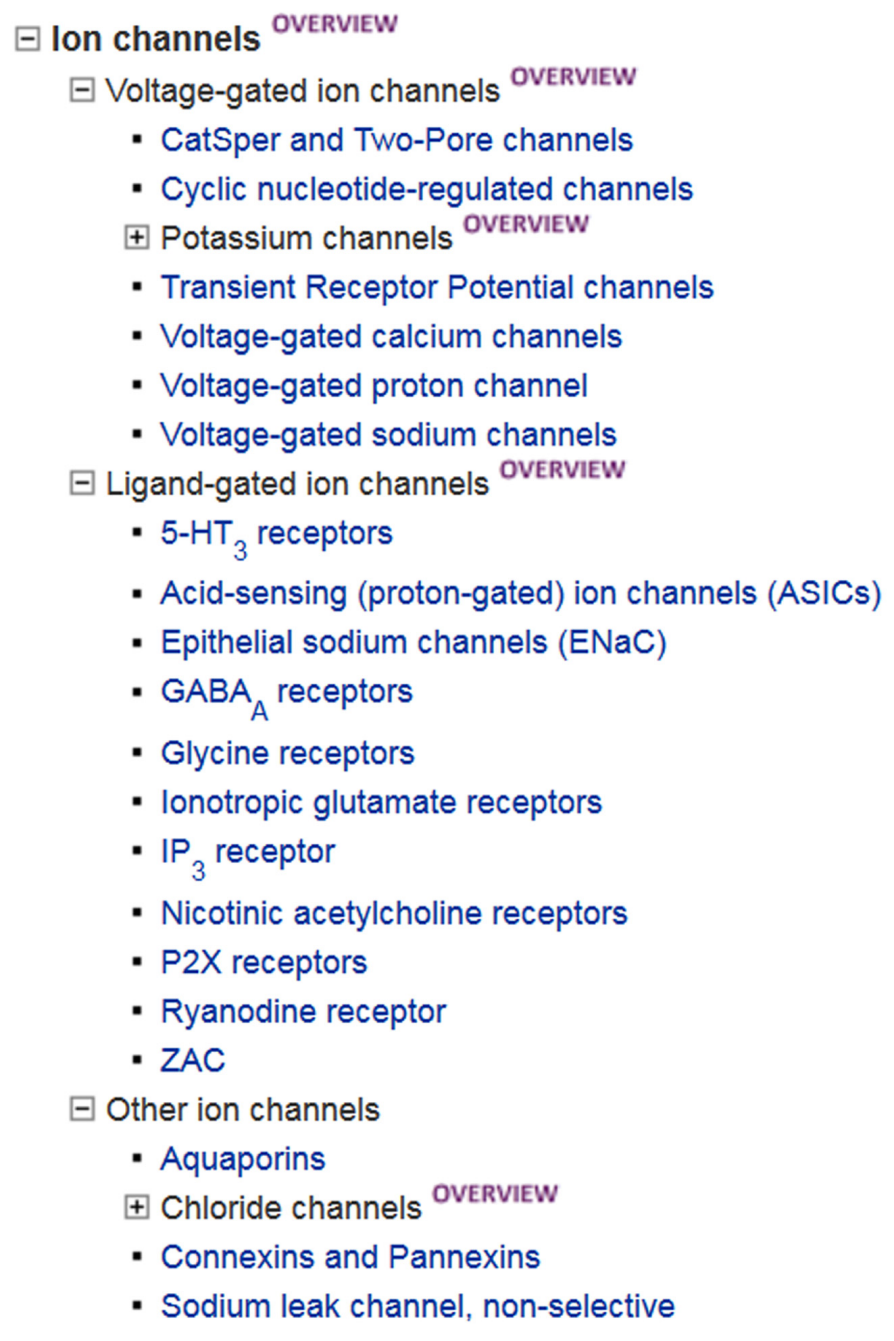
Hierarchical listing for the ion channel families and subfamilies.

Since our 2014 NAR publication, expansion has focused on new families that have a significant density of ligand mappings and drug target interest. We have not yet included all 523 proteases (as counted in human Swiss-Prot by the intersect of hydrolase function with a MEROPS (7) cross-reference), opting instead for a ligand-driven expansion in the first instance. For the kinome, all 539 entries (selected by our NC-IUPHAR kinase subcommittee) were pre-loaded because of the inclusion of matrix screens (see below) and proposals to complete tool compound coverage (8,9). We continue to add ligand mappings for both these large target classes (supported by the NC-IUPHAR protease and kinase subcommittees). Users can access data for each of the nine target classes in Table 1 *via* the GtoPdb website. The ion channel hierarchy is shown as an example (Figure 1). Where possible we adhere to the HGNC (10) Gene Families Index (http://www.genenames.org/cgi-bin/genefamilies/), but there are instances where the NC-IUPHAR classification deviates from these (e.g. catalytic receptors).

In the database, the term ‘target’ includes verified targets for the MMOAs for drugs used to treat human diseases, newer receptor-ligand pairings judged to be credible by a dedicated NC-IUPHAR subcommittee (11), and human targets identified by orthologue activity mapping where only non-human binding data are available. Examples of the latter category include the first generation of approved Angiotensin-converting enzyme (ACE) inhibitors, such as moexiprilat, for which only the rabbit protein has documented quantitative pharmacology. In addition, the database contains the targets of undesirable ligand interactions (sometimes termed ‘anti-targets’), for example the HERG channel, K_v_11.1 (*KCNH2*) as a liability target for cardiac toxicity from the withdrawn drug terfenadine. Target capture also extends to emergent targets—proteins that do not have sufficient validation data to be considered *bona fide* therapeutic drug targets, but are nonetheless being investigated to both establish their normal function and possible disease involvement. Cathepsin A (*CTSA*) is an interesting recent example, because not only is compound 8a [PMID 22861813] being explored to treat cardiac hypertrophy, but also an approved antiviral drug telaprevir is now being investigated for repurposing as a Cathepsin A inhibitor.

### Target statistics

**Figure 2.**
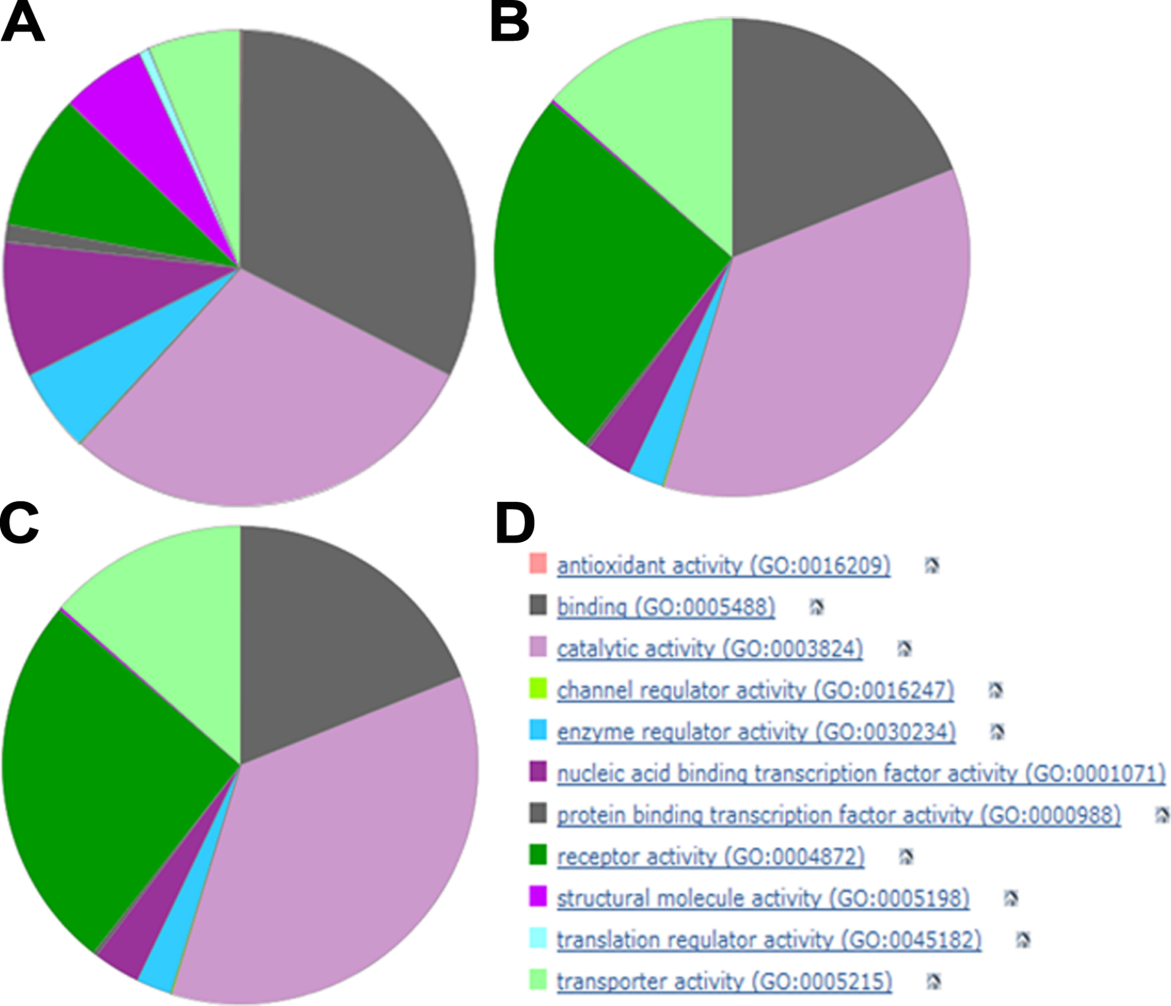
High level Gene Ontology (GO) functional categories for three sets of human proteins. Set **A** was generated from the total proteome of 20,204. Set **B** represents the 1228 targets with quantitative ligand binding data in GtoPdb. Set **C** represents the 554 targets where at least one approved drug is included in the ligand binding data. Panel **D** provides the colour key to the top-level GO categories. The charts were generated by loading Swiss-Prot IDs from the protein sets into the PANTHER Gene List Analysis Tool (55).

One of the benefits of our recently enhanced curation is that it enables more detailed exploration of statistics of database content. This gives us a detailed overview of the database and allows us to compare it with other resources, to communicate results to users and funders, to measure progress and identify areas for future expansion. Target-centric examples of such statistics are shown in Figure 2.

While the top-level GO categories are relatively coarse and not exclusive (e.g. some proteins are under both binding and enzymes), they provide a straightforward visual assessment of differences between protein sets. Not surprisingly, the curated set of ligand-binding targets (set B in Figure 2), compared to the whole proteome (set A in Figure 2), is enriched for receptors, enzymes and transporters. By selecting only targets of approved drugs (set C in Figure 2) we see a similar pattern to set B, but a proportional increase of both receptors and channels at the expense of enzymes. These results provide detailed insights into relationship distributions as well as the current state of pharmacology and therapeutics. Such analyses can be extended by many levels of detail to include other approaches (e.g. UniProtKB indexing and cross-referencing).

### Ligands

In the GtoPdb context, the term ‘ligand’ is used mostly for small molecule-to-large molecule interactions but it does extend to selected protein-protein interactions (e.g. cytokines-to-receptors or antibodies-to-cytokines). Interactions are selected for curation because they meet most of the following criteria:

1. mediated by direct binding (i.e. thermodynamically driven);
2. interaction is specific (i.e. reported cross-reactivity does not indicate promiscuity);
3. have experimentally measured quantitative binding-related results;
4. modulate the activity of their targets with biochemical consequences;
5. have distinct pharmacologically-relevant effects (even if unknown MMOAs);
6. related to drug discovery research for human disease;
7. published descriptions are resolvable to molecular structures;
8. reported *in vitro* potencies are judged to be mechanistically relevant to *in vivo* pharmacology (i.e. usually below 1 μM).

Our classification is divided into endogenous ligands (e.g. metabolites, hormones, neurotransmitters and cytokines) and exogenous ligands (e.g. drugs, research leads, toxins and probe compounds). Since our 2014 publication, the increase has been mainly driven by target-centric expansion (i.e. *via* target-to-ligand curation), but we have also focused on the following ligand selections (i.e. ligand-to-target curation) because of strong user interest:

- approved drugs;
- clinical development candidates (typically Phase 1 or beyond);
- approved or clinically-trialled monoclonal antibodies (i.e. with International Nonproprietary Names (INNs));
- compounds from repurposing initiatives (e.g. the National Center for Advancing Translational Sciences and Medical Research Council);
- epigenetic and kinase probes from the Structural Genomics Consortium;
- representative compounds directed against reported Alzheimer's Disease (AD) targets;
- R&D portfolio compounds associated with journal papers and/or repurposing documentation from selected companies (e.g. AstraZeneca);
- new human Protein Data Bank (PDB) (12,13) ligand structures;
- review articles with high density of relevant ligand-to-protein relationships;
- ligands highlighted in new papers of particular interest but outside the categories above, to which we were alerted by NC-IUPHAR subcommittee members, the GtoPdb team or Twitter notifications.

Ligand lists are displayed in nine categories and can be accessed at http://www.guidetopharmacology.org/GRAC/LigandListForward. Current counts for each of these categories are provided in Table 2.

**Table 2.** Ligand category counts. SID refers to the PubChem Substance Identifier and CID the PubChem Compound Identifier

Ligand classification	Count
Synthetic organics	5055
Metabolites	582
Endogenous peptides	759
Other peptides including synthetic peptides	1222
Natural products	234
Antibodies	138
Inorganics	34
Approved drugs	1233
Withdrawn drugs	67
Ligands with INNs	1882
Isotopically labelled ligands	593
PubChem CIDs	6037
PubChem SIDs	8024
Total number of ligands	8024

### PubChem content

**Figure 3.**
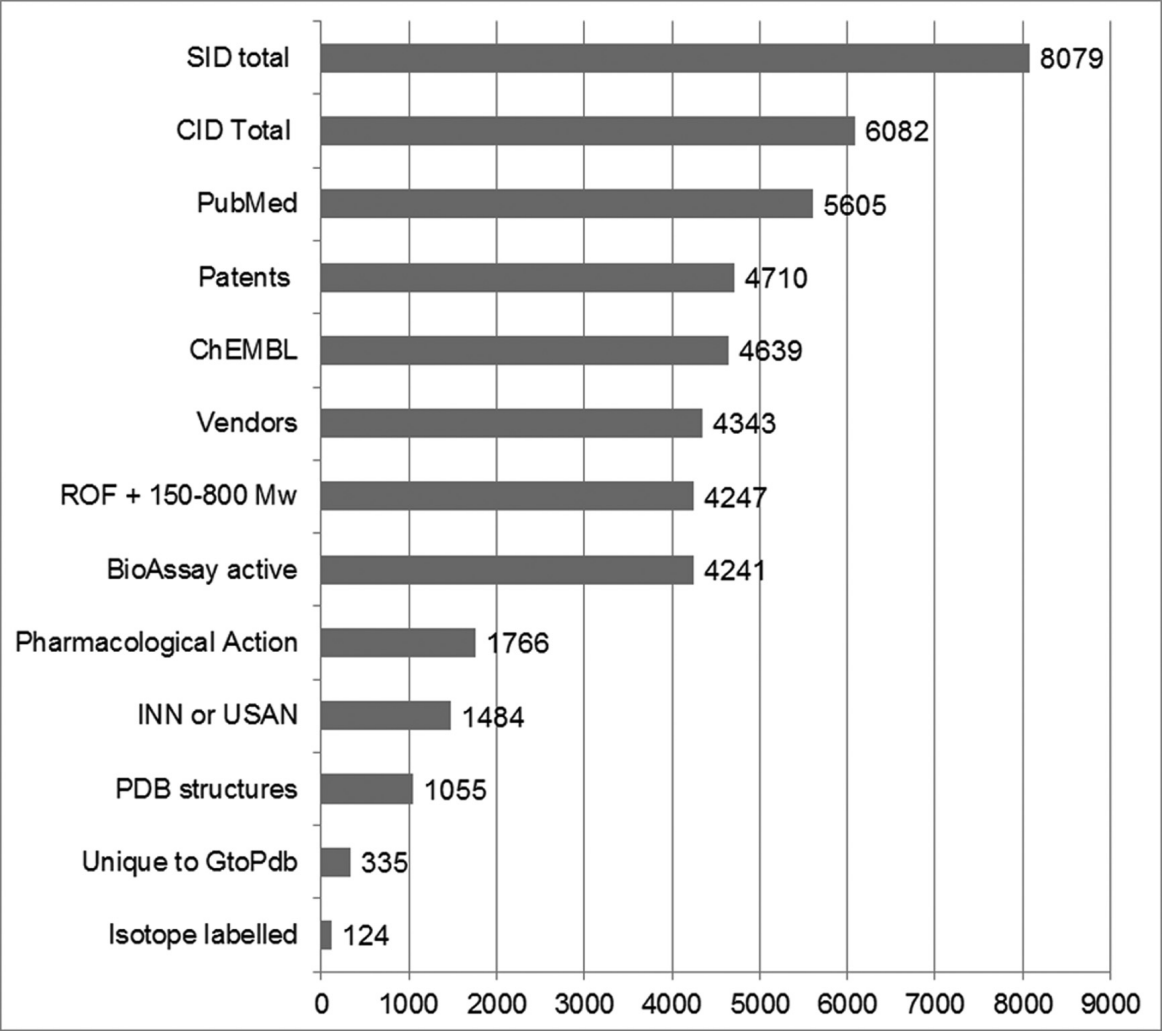
PubChem intersects. Figures were obtained *via* the PubChem interface using mostly pre-existing indexing. The exceptions are custom selects (described below) for patents, INN or United States Adopted Names (USAN) and Lipinski Rule-of-Five (ROF) + 150–800 Mw. With the exception of the SIDs (Row 1) intersects are CID counts. These queries were executed at the beginning of September 2015 when the PubChem CID total was 60.8 million and our own SIDs from release 2015.2 had been processed.

Since our 2014 publication, we have adopted the PubChem Compound ID (CID) as our primary small-molecule identifier and we refresh our own ligands as PubChem Substance Identifiers (SIDs) for each release. This means we (and, importantly, anyone else) can generate a detailed analysis of our content (14,15). This provides uniquely high-resolution breakdowns for a wide range of categories, sources and properties, and these can be selected for their chemical and/or biological annotation types. The distributions for a selection of these are shown in Figure 3.

We aim to complete a PubChem re-submission within two weeks of our public releases. Our SIDs are then merged into CIDs according to the PubChem chemistry rules (Figure 3, Rows 1 and 2). The excess of SIDs over CIDs reflects those SIDs that do not have chemical structure representable in SMILES format (i.e. cannot form CIDs). Most of these are large peptides or small proteins but also include our antibody entries. We also revise a small number of entries between our release re-submissions. As expected, since it is our major curation source, over 90% of structures can be linked to a PubMed ID either *via* Entrez or ChEMBL (Figure 3, Row 3). For patent extraction matches, a filter was made from the three PubChem sources (IBM, SCRIPDB and SureChEMBL) that use automated Chemical Named Entity Recognition and include patent document numbers in the CID records. At 78% (Figure 3, Row 4), this is much higher than in 2013 due to the increase in patent chemistry in PubChem (16). While our matches overlap ChEMBL by 76% (Figure 3, Row 5), we have 1361 structures not in this source. The proportion of CIDs having a match to at least one chemical vendor SID has risen to 72% (Figure 3, Row 6). Another filter was used as the Lipinski Rule-of-Five (ROF) with an extended molecular weight (Mw) range. Thus, 70% of our structures are inside this medicinal chemistry property ‘sweet zone’ that encompasses both drugs and leads (Figure 3, Row 7). The BioAssay matches (Figure 3, Row 8) coincide with the ChEMBL count at 70% but are complementary because of extended connectivity to data sets from the Molecular Libraries initiative (3).

Just 30% of our CIDs have a match to the MeSH term ‘Pharmacological Actions’ (Figure 3, Row 9), which means the compound has been assigned pharmacological *in vivo* mechanisms of action by MeSH curators based on the paper in which it was reported. This total is surprisingly low and indicates a capture gap for this MeSH category. We recorded a 25% intersect of our compounds with the 10,939 CIDs retrieved by the query ‘INN (or) USAN’ which represent non-proprietary names for either approved drugs or failed clinical candidates (Figure 3, Row 10). The number of GtoPdb ligands with a match to PDB structures is 17% (Figure 3, Row 11). The 335 CIDs unique to us in PubChem (Figure 3, Row 12) include compounds extracted from documents, either before they might appear from other submitters, or curated from journals not extracted by other sources. The designation of radiolabelled ligands in GtoPdb presents a curatorial challenge because for 467 entries, the publications we have curated do not specify the exact substitution position for the radioisotope. Consequently, we only have 118 CIDs (Figure 3, Row 13) where this was defined by the authors. Because of strong interest in these compounds as pharmacological tools, we have had to re-use the unmodified structure (thereby effectively generating a duplicate) in order to explicitly link the radiolabelled compound names to the published experiments.

A caveat associated with the statistics in Figure 3 arises from the numbers being CID ‘exact match’ results (i.e. equivalent to a full InChI-to-InChI match). For individual cases, users can either use the PubChem ‘same connectivity’ operator to reveal structures with the same carbon skeleton or, from our pages, execute a Google search with either the full InChIKey or just the core layer. Thus, most commonly in terms of salt forms or different stereoisomer representations of the same core structure, our CIDs may have additional matches (i.e. be the same compound in pharmacological terms) in source entries other than those counted above (but with different CIDs).

### Interaction mapping

Quantitative ligand-to-protein interaction mappings constitute the core of the database. Curated relationship data across all targets is shown in Table 3. The total number of references in GtoPdb has reached 27880, a figure that includes the many target-specific references we also capture. Most (98%) have PubMed IDs but we include a few other reference types judged to be sufficiently provenanced. These include journals not indexed in PubMed, patents, slide sets, meeting abstracts, confirmed PubChem BioAssays and pharmaceutical company open information sheets for (unpublished) repurposing candidates.

**Table 3.** Interaction counts. Primary target indicates the dominant MMOA

Interaction type	Count
Targets with ligand interactions	1505
Targets with quantitative ligand interactions	1228
Targets with approved drug interactions	554
Primary targets with approved drug interactions	312
Ligands with target interactions	6796
Ligands with quantitative interactions (approved drugs)	5860 (738)
Ligands with clinical use summaries (approved drugs)	1724 (1231)
Number of binding constants	44691
Number of binding constants curated from the literature	13484

### Kinases

In 2013, we added three published sets of results from cross-screening of kinase panels, to extend data for this important target class (17–19). The cumulative set of 406 kinases x 230 ligands includes 158551 data points for users to inspect. An example from the imatinib entry is shown in Supplementary Figure S1.

The constitutive problem with surfacing panel screens in a database is that the assays are balanced to produce mostly negative results (i.e. compounds will be predominantly inactive at the threshold tested). In addition, the *Millipore* and *Reaction Biology* sets measure only percentage-activity-remaining at fixed concentrations, rather than dose-responses. For this reason, we separate the kinase panel results from the curated literature values (typically selected as active IC_50_ or K_i_ rather than K_d_) in our data model and mapping statistics. Users can see both in the web display (Supplementary Figure S1; note that only the top 10 targets in each of the screens are displayed on the ligand page, with the option to view the full set). As a cross-check, we determined that 68 kinases in the *DiscoveRx* panel had a pAct (pK_d_) value for a panel ligand at 7 or above (i.e. 100 nM or less). We had independently curated literature inhibition values for each of these 68 (but not necessarily for the same ligand and/or assay conditions) indicating there were no high-potency kinase panel results for which we did not also have curated data values.

### Single versus multiple versus complex targets

As explained above, our capture of ligand-target relationships is founded on citable activity data that define pharmacologically significant molecular interactions. We recently enhanced our mapping precision by introducing the concept of a primary target, identified with a tag, when the publication record indicates that drug or lead has been optimised for a single target. By implication, the *in vitro* MMOA is likely to be causative for observed therapeutic effect *in vivo* (e.g. the effect of perindoprilat in lowering blood pressure is due to its substrate-competitive binding potency (IC_50_) of 1 nM against ACE). Nonetheless this assumption has to be caveated where *in vivo* target validation data are still pending (e.g. *via* mouse KO and/or a clear genetic disease association). The curator-assigned ‘primary target’ tags delineate a concise drug-to-target set of 312 human proteins for approved drugs.

We are well aware of the challenges of setting curatorial stringencies for structure-to-activity-to-target mapping (20). One aspect of increasing importance is polypharmacology, where evidence suggests that clinical efficacy is mediated by multiple MMOAs. The simplest examples are drugs designed as dual inhibitors, such as fasidotrilat (an antihypertensive agent that acts on both ACE and NEP ) where data support our assignment of two primary target relationships to the ligand. The situation is more complex for kinase inhibitors where *in vitro* data indicate that certain clinically successful inhibitors have polypharmacologic MMOAs (9). Nonetheless, for relationship curation it remains difficult to define exactly which binding results are causatively relevant or if their capture is useful for GtoPdb data mining. For this reason, we capture non-primary interactions but do not tag them explicitly as ‘secondary targets’. We thus generally leave the interpretations of significance (e.g. efficacious polypharmacology, off-target interactions or side effect liabilities) open. An example here would be bosutinib which has 24 curated interactions: only one of these is tagged as primary, while the others are recorded for user interpretation. However, in cases where the pharmacological significance of off-(primary) target binding data is clear we will add a curators comment.

For complex targets, we have again taken a parsimonious approach (in line with the primary target concept) in mapping to the minimal, rather than maximal, number of proteins, to increase data mining precision (21). Examples here include the approved proteasome inhibitor bortezomib and the clinical candidate gamma-secretase inhibitor begacestat. We have mapped the former just to one subunit, beta type, 5 protein, for which there is evidence for direct binding of the drug, rather than adding the 43 distinct components of the proteasome endopeptidase complex into our relationship matrix. Analogously, the latter inhibitor is mapped just to presenilin 1 (*PSEN1*) rather than all five components of the gamma secretase complex.

### Relationship distribution

The recent expansion phase has been predominantly target-centric. Consequently, the distribution of quantitative mappings to targets has become more long-tailed. As expected, the average ligands-per-target fell from 11 to 8 as the target total extended from 844 to 1401. Our statistical analysis of this distribution (results not shown) highlighted important aspects. One of these is the need to control the occupancy at the top end of the distribution. As two examples, the dopamine D_1_ receptor has 19 agonists and 15 antagonists that include 17 approved drugs, whereas the kinase VEGFR-2 (*KDR*) has 54 inhibitors, including 14 approved drugs (two of which are antibodies). While we have not introduced an upper limit for ligands-per-target, we would clearly impose a high threshold (based on pharmacological significance) in these cases, before adding new ligands. This contrasts with targets in the tail of the distribution where the threshold for adding new ligands remains low. For example, transmembrane protease, serine 6 (*TMPRSS6*) only has a single inhibitor (inhibitor 1 [Colombo et al., 2012]) so far, but, because the protein has a loss-of-function Mendelian disease association with iron deficiency anaemia, new functional probes may be published. The ‘tailing’ effect is also manifest in our numbers of 207 single-ligand targets in 2013 expanding to 637 in 2015.

Notwithstanding our emphasis on establishing connectivity for data mining, we also capture compounds with important pharmacological effects where the therapeutic MMOA is unknown or remains equivocal. Perhaps the best known approved drug example is lithium, but we also have research compounds where curator comments indicate a phenotypic read-out and/or pathway-mapping as a partial MMOA (e.g. CCG-1423).

### Entity growth

The figures in Table 4 record recent increases in entities and selected attributes.

**Table 4.** Content changes since our 2014 publication (6). Only those major categories that could be normalised for comparison between 2013 and 2015 are included

	Oct 2013	2015	Percentage increase
Target protein IDs	2485	2761	11
Ligands total	6064	8024	32
Approved drugs	559	1233	121
Antibodies	10	138	1280
Peptides	1776	1981	12
Synthetic small molecules	3504	5055	44
PubChem SIDs	3107	8024	158
PubChem CIDs	2694	6037	124
Binding constants	41076	44691	9
References	21774	27880	28

**Figure 4.**
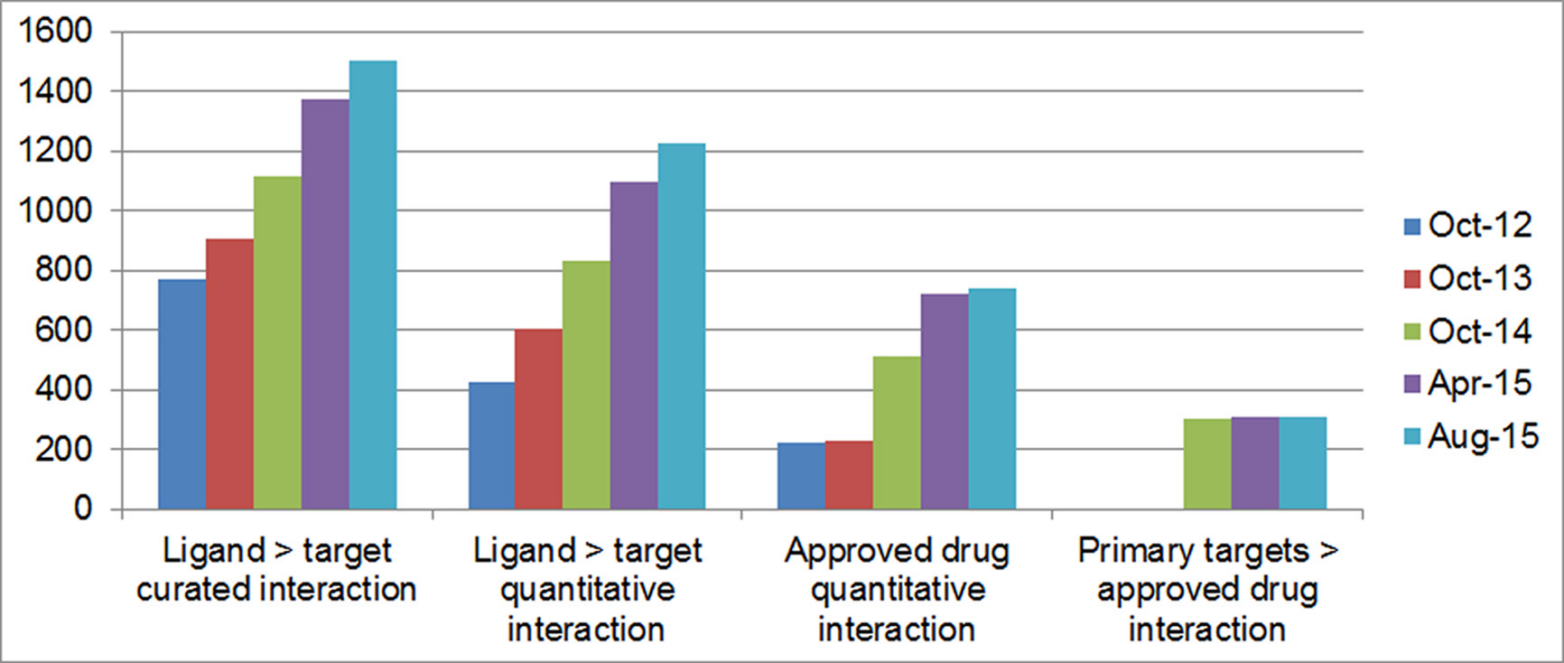
Relationship growth since 2012. The first (left-most) chart shows the number of targets with curated ligand interactions while the second chart includes only those targets that are supported by quantitative data. The third and fourth charts show the number of approved drugs with data-supported targets and those that may be considered primary targets, respectively.

Since the last publication, the largest entity-type increase has been antibodies. The next categories, in order of increase, are approved drugs and PubChem entries. We have added new CID links to older entities (i.e. more of the structures we already had are now assigned to CIDs). We have also plotted the relationship metrics for a spread of release versions, including the one preceding our 2014 publication (Figure 4).

Three of the four relationships show steady growth but the classification of primary targets of approved drugs shows a flattening off. This was expected because the curation of most of these target relationships (for at least one approved drug) had been largely completed by the end of 2014. Approved drug curation, including new approvals directed against existing targets, continued in 2015 but the number of new protein targets mapped was very low.

## CURATION ENHANCEMENTS

### Strategy

In collaboration with our target-family subcommittees, we have enhanced our curation procedures, because they are the primary determinant of database value. Crucially, this includes deciding what to leave out as well as include, and we have introduced more stringent filtering to maximise the utility of our relationship matrix. However, while we make use of established ontologies and terminologies where possible (e.g. see the disease section below), we do not apply rigid rules for content capture. We instead make extensive use of curators’ comments that allow us to bridge between structured annotations (i.e. indexed in the database) and the flexibility of unstructured text. For users, we can thus specify new (or low frequency edge-case) relationship types *via* cross-pointers that are not formalised in the current schema (we may decide later to accommodate these *via* new structured indexing, if enough examples and an external terminology consensus appear). An illustration of this is where we add ‘repurposing’ to ligand comments. The term is used rather loosely in the literature but a simple text query retrieves a list of compounds, with particular interest to many users, where we judged the mention in a publication as relevant.

Another manifestation of curatorial flexibility is that we will add ligands from the earliest reports of chemical modulators for a novel target (possibly patent-only), even if these are of such low potency and/or specificity as would be unpublishable for a well-characterised target (e.g. surrogate ligands for orphan receptors). We will add superior ligands as they are published, but do not typically remove older ligands with cited references. Another unique strategic aspect is the undertaking of rolling updates by the subcommittees. This includes not only adding context to new relationships, but also reviewing their physiological and molecular aspects. Indeed, many of our users come to the database to learn about target proteins of interest in terms of family relationships and roles in different settings.

### Approved drugs

Our grant objectives include annotating the targets of approved human medicines (i.e. currently not anti-infectives). However, the task is complicated by variation in database molecular structures for approved drugs (22). For this reason, we have chosen a consensus approach whereby we select the PubChem CID supported by the most submitters (i.e. has the SID ‘majority vote’). We realise this approach is not infallible, but it does have pragmatic utility. Specifically, an exact chemical structure match between a majority of sources (at least some of which are manually curated) is more likely to be right than wrong. An example is provided by vapiprost where the CID 6918030 we have selected as (Z)-7-[(1R,2R,3S,5S) is supported by 13 SIDs, including that of ChEMBL, the Food and Drug Administration (FDA) Substance Product Labelling entry, and is concordant with the INN document as well as the CAS Registry No. 85505–64–2. The alternative (E)-7-[(1R,2R,3S,5S) form is represented by nine SIDs merged into CID 6436588. The PubChem ‘same connectivity’ relationships records 13 CIDs (i.e. 11 additional ones) with various permutations or absences of the stereo specifications.

We have reached a current total of 1222 approved drugs (including antibodies) for which we have been able to curate drug-to-target relationships, and this covers new FDA approvals to 2Q 2015. This is lower that we might expect, but there is no agreement on what the approved drug count should be at the molecular level (sources indicate anywhere between 1200 and 1600). This anomaly emphasises the complexities associated with the concept of drug structure ‘correctness’. We use curatorial stringency to limit, as far as possible, consequences of different structural representations of the same drugs and associated splitting of activity mappings.

Two examples illustrate this. Since drugs can have many salt forms, we typically select the parent CID for our target and activity mappings. This is not only because this usually corresponds to the INN name-to-structure mapping, but for *in vitro* experiments the parent ion is usually the active moiety. However, records in PubChem BioAssay and USAN designations often map to salt forms. A second example is where an approved drug is an enantiomeric mixture (that does not interconvert *in vivo*), but assay data can be mapped to three different molecular representations (i.e. both the R and S isomers and the mixture or ‘flat’ form). In this case, we assign the drug tag to the mixture and map data to this. We then add cross-pointers to the CIDs for the R and S if data have been specifically reported and mapped to them. Well known examples are omeprazole as the mixture and esomeprazole as the S isomer, as separately approved drugs. We include both withdrawn and discontinued drugs (the latter being generally superseded by newer drugs) to maximise our capture and cheminformatic analysis of drug sets. The terms are not exclusive (i.e. a drug can be tagged as both approved and withdrawn) but these can be filtered out of queries if necessary.

For a number of reasons, we will not attempt to capture all molecular entities approved for human use. The main reason is because the database is focused on quantitative molecular pharmacology, captured as a ligand-target relationship matrix to facilitate data navigation and mining. It is thus not a pharmacopeia-type compendium (of which many are available), because many substances approved for medicinal purposes would negatively impact the precision of our database if we mapped-in their molecular interactions as ‘drugs’. We therefore exclude simple molecules such as acetic acid, ethanol, urea and common inorganic salts. We also omit nutraceuticals that are principally metabolites (e.g. we do not target-map the DrugBank ‘approved drug’ entry for NADH that lists 144 targets).

### Patent exploitation

**Figure 5.**
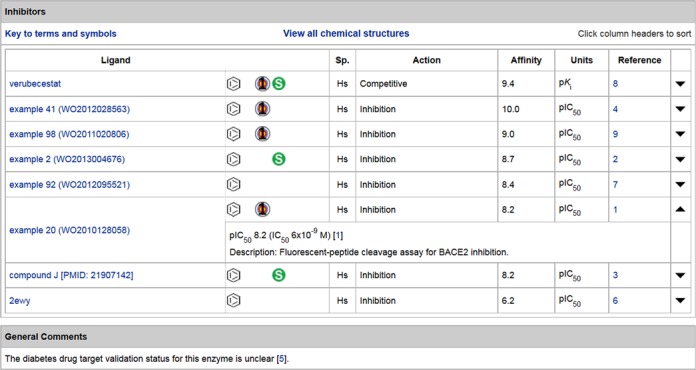
Inhibitors table from the detailed view of the BACE2 target entry, with the inclusion of five lead compounds from patents.

While our main extraction source remains the peer-reviewed literature, we increasingly exploit patents for their unique data content in particular cases. This has become easier because of the ‘big bang’ in the recent open availability of over 15 million patent-extracted chemical structures in several large PubChem sources (16). We cite medicinal chemistry patents in two circumstances: (i) where potent and selective ligands are patent-only or (ii) where documented structure activity relationships (SAR) are particularly complementary to those from published articles from the same team (e.g. because many more analogues have quantitative data and synthesis descriptions). We generally link to patents only from those pharmaceutical companies and academic institutions with an established medicinal chemistry reputation. An example of the value of patent data is shown in Figure 5 for beta-site APP-cleaving enzyme 2 (*BACE2*).

The BACE2-selective inhibitors claimed specifically as potential anti-diabetes compounds are, as far as we can determine, the only public database instantiation of these activity mappings (23). In this context, it is important to note that ChEMBL does in fact map 574 compounds to human BACE2 (target ID CHEMBL2525). However, these are all BACE1 inhibitors extracted from journal articles that have included BACE2 cross-screening results, since the first paper specifying the use of BACE2 inhibition for diabetes used a single BACE1 inhibitor and no medicinal chemistry papers have described BACE2-selective inhibitors. Thus, the chemistry is captured in SureChEMBL and GtoPdb, but not ChEMBL.

We have also been able to exploit patents as a source of both primary sequence and target binding data. This has been particularly useful for monoclonal antibodies and exogenous therapeutic proteins or peptides where these data may be absent from journal articles. In these cases, the patent sequence databases provide the entry point and we can also add cross-references to the UniParc records (24).

## DISEASE ONTOLOGY AND CLINICAL VARIANTS

**Figure 6.**
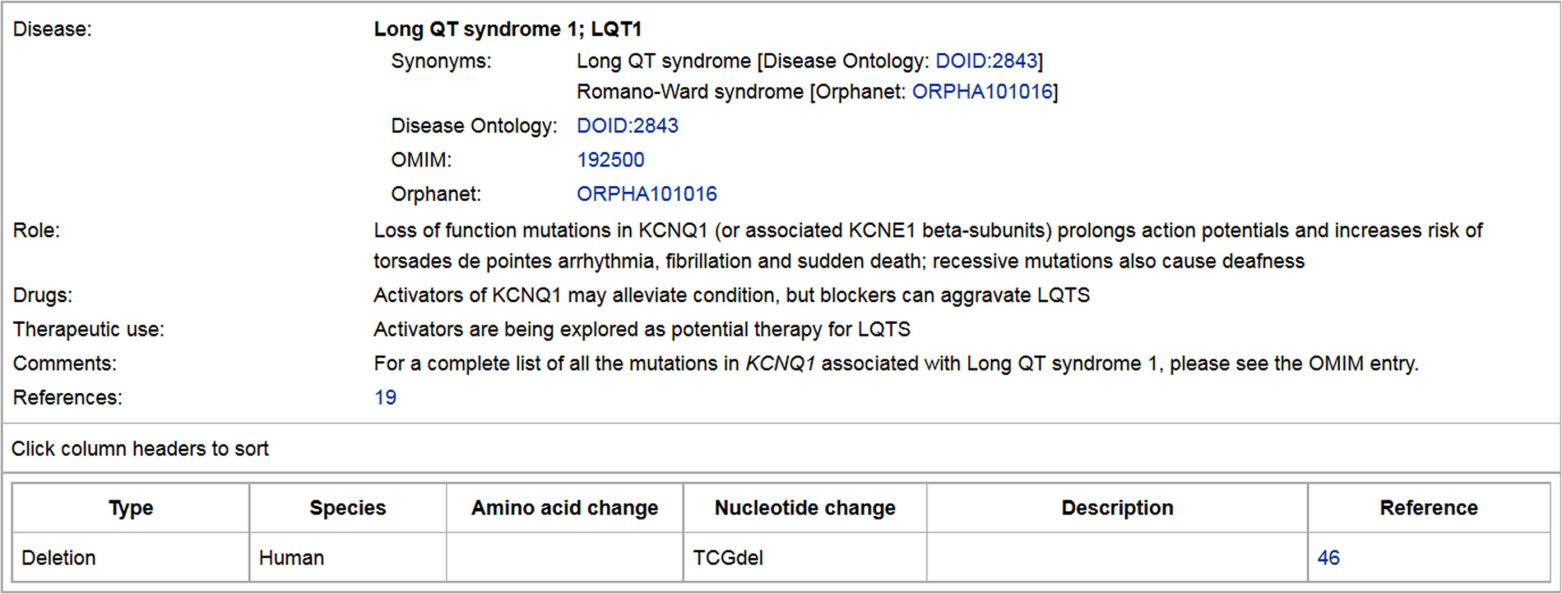
Clinically-Relevant Mutations and Pathophysiology for Kv7.1.

**Figure 7.**
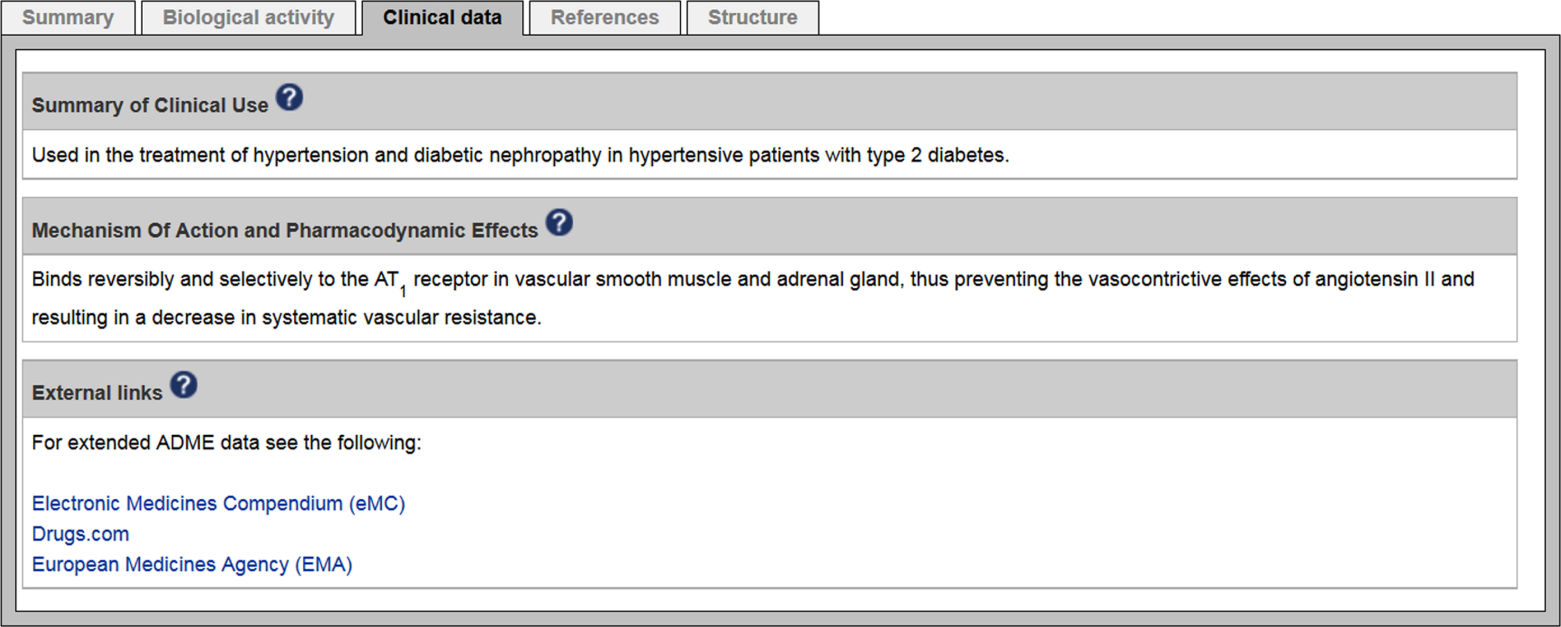
Clinical data summary tab for the approved drug telmisartan.

Another major effort since our 2014 publication has been the review and expansion of target-linked diseases and associated mutations (Figure 6). We used the tool ‘ZOOMA’ (http://www.ebi.ac.uk/fgpt/zooma/index.html) to map our disease names to Disease Ontology (25) and Orphanet Rare Disease Ontology (http://www.orphadata.org/cgi-bin/inc/ordo_orphanet.inc.php) terms, and now use standardised disease names that, wherever possible, are linked to synonyms (which may include more general names for specific subtypes) and entries on the Orphanet (26) and OMIM (http://omim.org/) websites. Disease Ontology terms are linked to the Ontobee browser (27) which provides contextual visualisation. Diseases are linked to targets *via* ‘pathophysiologies’ which describe the role of the target in the disease, possibly including drugs and side effects, as well as disease-causing mutations. Mutation descriptions have also been standardised within GtoPdb. Future releases will link drugs to diseases *via* the clinical data tab (Figure 7) and provide new target-disease-drug navigation options. This will not only allow users to browse and search using disease names but also enable us to present disease pages containing lists of associated targets and ligands. We also intend to review our listings of single nucleotide polymorphism (SNP) variants, many of which are disease-associated.

## WEBSITE FEATURES

The following description includes some basic aspects for context, but focuses on the most important features added since the previous report. We have improved our help documentation and tutorials. This now includes a substantial set of frequently asked questions (FAQs) at http://www.guidetopharmacology.org/faq.jsp) that inform users on new features and data types. Enhancements have been made to the search tools to improve user experience of the website. The quick search box at the top right of every page and the advanced search pages for targets and ligands now include autocomplete functionality for target, target family and ligand names. Users are able to click on the matched name and go directly to the corresponding database page. We have also added support for the recognition of special characters such as Greek letters found in target names (e.g. δ opioid receptor). Our ligand structure search tool uses the JavaScript chemical editor Marvin JS (ChemAxon Limited, Hungary), which replaces the Java applet version and offers cross-platform compatibility including for tablets and mobile devices. Searches now cover more database fields which allows, for example, searches by disease name to retrieve associated targets and ligands.

As well as providing a variety of ways to search the database (e.g. name, keyword, database identifier or ligand structure), users can browse target and ligand lists according to their biological or chemical classification. To deal with the increasing size of the database and intersecting classifications for some targets (e.g. EC 3.4 and protease) we have introduced a hierarchical organisation. Targets are grouped into families and subfamilies and visualised as a navigable HTML tree with expandable and collapsible nodes (see Figure 1 for example). Each family has a linked database page including an overview, background reading and details of subfamilies or individual family member proteins. Alternatively, users may browse lists of ligands organised by chemical class or drug approval status. We have introduced a new category of labelled ligands for those with radioactive incorporation or a fluorescent moiety. Labelled ligands are also indicated within bioactivity data tables using a new symbol. We have also added two other new symbols to bioactivity tables to indicate where the ligand is an approved drug, and (as described above) where the target can be considered the primary data-supported target of that ligand. Furthermore, the information curated in support of new interactions has been expanded to include affinity data and details of the assay used, accessible in the bioactivity table by clicking on the arrow at the right (e.g. see the entry for ligand ‘example 20 (WO2010128058)’ in Figure 4).

Our grant mandate to curate the MMOAs of approved drugs and clinical candidates has led to the introduction of various new features on the ligand pages. A new ‘clinical data’ tab provides summaries of clinical use, MMOA, as well as absorption, distribution, metabolism and excretion (ADME) data (Figure 7). Drug approval status is indicated along with the FDA and European Medicines Agency (EMA) first approval dates (a small number of drugs approved only in Japan are also included). INN compounds now have on-the-fly name searches of PubMed titles, abstracts and clinical trials. In addition, small molecules have InChIKey searches of Google for exact or backbone chemical structure matches to many databases and chemical vendors (28).

## CONNECTIVITY COLLABORATIONS

We manually curate out-links to other databases that we judge as having utility for a significant fraction of users. This applies both for navigation and computational mining across linked data. For this reason, we continually review out-links and monitor the status of reciprocal in-links (but note there may also be in-links of which we are unaware). We also maintain a tradition of collaborative networking with most of these resources, with inter-team contacts often initiated at conferences and/or NC-IUPHAR meetings. A selection of those collaborative interactions that have had direct technical consequences for connectivity and with whom we have arranged reciprocity, is outlined in Table 5 (more of these are pending and we are open to new engagements).

**Table 5.** Examples of links where we have direct interactions with the database teams

Resource	Connectivity Comments	Reference
BindingDB	Comprehensive ligand-target database, we now cross-reference selected patent extractions from this source	(43)
ChEMBL and UniChem	Inclusion of our target protein pointers and a ChEMBL look-up for our ligand entries loaded in UniChem	(2,44)
DrugBank	Target cross-references and chemical ontology connection *via* an API	(45)
ESTER	Alpha/beta hydrolase cross-references	(46)
GeneCards	Gene expression and functional data aggregator	(47)
GPCRDB	Specific pointers to their detailed features, curation of mutations, sequence display toolbox and residue numbering system	(48)
GUDMAP	Links to proteins involved in GenitoUrinary (GU) tract development	(49)
HGNC	Long standing and frequent interactions on target family nomenclature issues	(10)
IMGT/mAb-DB	Pointers to provenanced sequences for clinical antibodies, target interactions, display tools and residue numbering system	(50)
MEROPS	Feature details, classification, ligand mapping, other protease-specific issues	(7)
neXtProt	Data and features additional to Swiss-Prot, semantic mining technology	(51)
NURSA	Detailed NHR information including transcriptome mining functionality	(52)
Orphanet	Unique rare genetic disease curation and disease term connectivity	(53)
PubChem	Covering aspects of chemical curation, drug naming and our submitted structures. Plans for future peptide and BioAssay Links	(4,14)
UniProtKB	Maintenance of our own selectable cross-references to proteins with quantitative interactions	(5)
Wikipedia	Updating, adding new target and ligand links, including filling in ‘chemistry boxes’	(54)

The overriding principle of collaborative cross-referencing is complementarity. The expansion of our interactions with GPCRDB during 2014/15 exemplifies this, since both resources have had historically overlapping engagement with the human GPCR repertoire (29,30). This has now evolved into collaborative strategic curatorial divergence, while at the same time offering users differential features for the 365 human Swiss-Prot IDs we have in common. In general, this is manifested by quantitative ligand mapping and major clinical variant collation on the GtoPdb side, complemented by the emphasis on sequence/structure relationships on the GPCRDB side, which includes data on engineered substitution variants. In addition, we are in the process of harmonising both our web services to make it easier for users to make entity and data joins between the two resources.

### Journal-to-database connectivity

We have three initiatives in this area. The first of these is the production of the *Concise Guide to PHARMACOLOGY* (CGTP), published online as a series of PDF documents (and in HTML) at two-yearly intervals as a supplement in the *British Journal of Pharmacology* (BJP). CGTP provides succinct overviews of families of drug targets in the form of a desktop reference guide. The first of these appeared in 2013 (31), with the second due for publication in November 2015. Thus, targets and ligands specified in the CGTP articles online are hyperlinked directly to the database records for users to navigate. To achieve this, the GtoPdb team and the CGTP editors collaborate with the Wiley publishers on what is, in effect, the automatic converting of (pre-tagged) sections of the database directly into the online CGTP PDF documents. The second initiative, also a collaboration with the BJP, involves marking-up tables of links (ToLs) for both regular papers and reviews (32). An example is a recent invited review on epigenetic pathway targets for the treatment of disease, which can be viewed at http://onlinelibrary.wiley.com/doi/10.1111/bph.12848/epdf (the ToLs are on the second page) (33). This exemplifies a ‘virtuous circle’ from our special relationship with the BJP and NC-IUPHAR. The invited review provided the curatorial starting point for the capture of new ligands and targets to populate the database and these were consequently surfaced as ToLs in the article. The third journal-to-database initiative is a logical extension of the previous two (32). This involves an updated version of the BJP instructions-to-authors that now includes recommendations on resolving the molecular identities of targets and ligands at the submission stage. The eventual surfacing of such ‘curation-ready’ manuscripts will expedite not only our capture of new database records, but also improved coverage for the ToLs.

## EXTERNAL PROFILE (NON-JOURNAL)

We continue to circulate our NC-IUPHAR newsletter that includes in-depth articles on various aspects of the database. In addition, we use various social media portals for outreach, updating existing users, announcing IUPHAR reviews and other publications and sharing upcoming meeting presentations. We also find these outlets valuable for occasional rapid technical exchanges with collaborating databases. Our blog (http://blog.guidetopharmacology.org/) includes detailed release descriptions, new features, and technical ‘how to’ items. One of us (CS) maintains an individual technical blog where GtoPdb topics are sometimes coupled by being briefly introduced in the GtoPdb blog but expanded on in the individual posts (http://cdsouthan.blogspot.com/). Our Slideshare account (http://www.slideshare.net/GuidetoPHARM) is used for sharing slide sets and posters with the community and has proved popular. Users will find that presentations include descriptions of content, mining approaches and utilities that extend beyond what is documented on the site. We have also added a set of generic slides which can be used by anyone presenting or teaching on GtoPdb. As another important part of an external profile we endeavour to regularly update our Wikipedia pages.

## CHALLENGES AND FUTURE DIRECTIONS

Recent publications continue to highlight challenges of operating in the intersection of bioinformatics and cheminformatics (20,21,34,35). One aspect we will be addressing arises from the statistical analysis of content. Not unexpectedly, this exposes some gaps and deficiencies. For example, we have a historical ligand-capture and information density bias towards GPCRs, ion channels and NHRs derived from the seed content in 2011 which his has persisted even though these targets are now outnumbered by enzymes (36). This legacy extends into the data structure. In the past, committees have input binding data from multiple references which has resulted in ranges being recorded in the older records for receptors and channels (e.g. somatostatin 1–28). However, extraction of multiple values from different papers could not be sustained for the recent phase of expansion because, as we move out into the target ‘long tail’, there are fewer independent measurements available.

Another challenge we want to address concerns the search space, formal representation and rendering (i.e. to provide informative visualisations) for our 1981 peptide ligands. These are too small for BLAST-type peptide searches and too large for Tanimoto-based small molecule searching. In addition, many have post-translational and/or synthetic chemical modifications. This means the linear primary sequence we include is incomplete as a structural specification (although we use IUPAC nomenclature for some modifications if sufficiently detailed in the papers). We have been testing algorithmic approaches that can ameliorate some of these problems, in particular HELM (37) and Sugar & Splice (NextMove Software, Cambridge, UK) and look forward to the launch of PubChem Biologicals towards the end of 2015.

**Figure 8.**
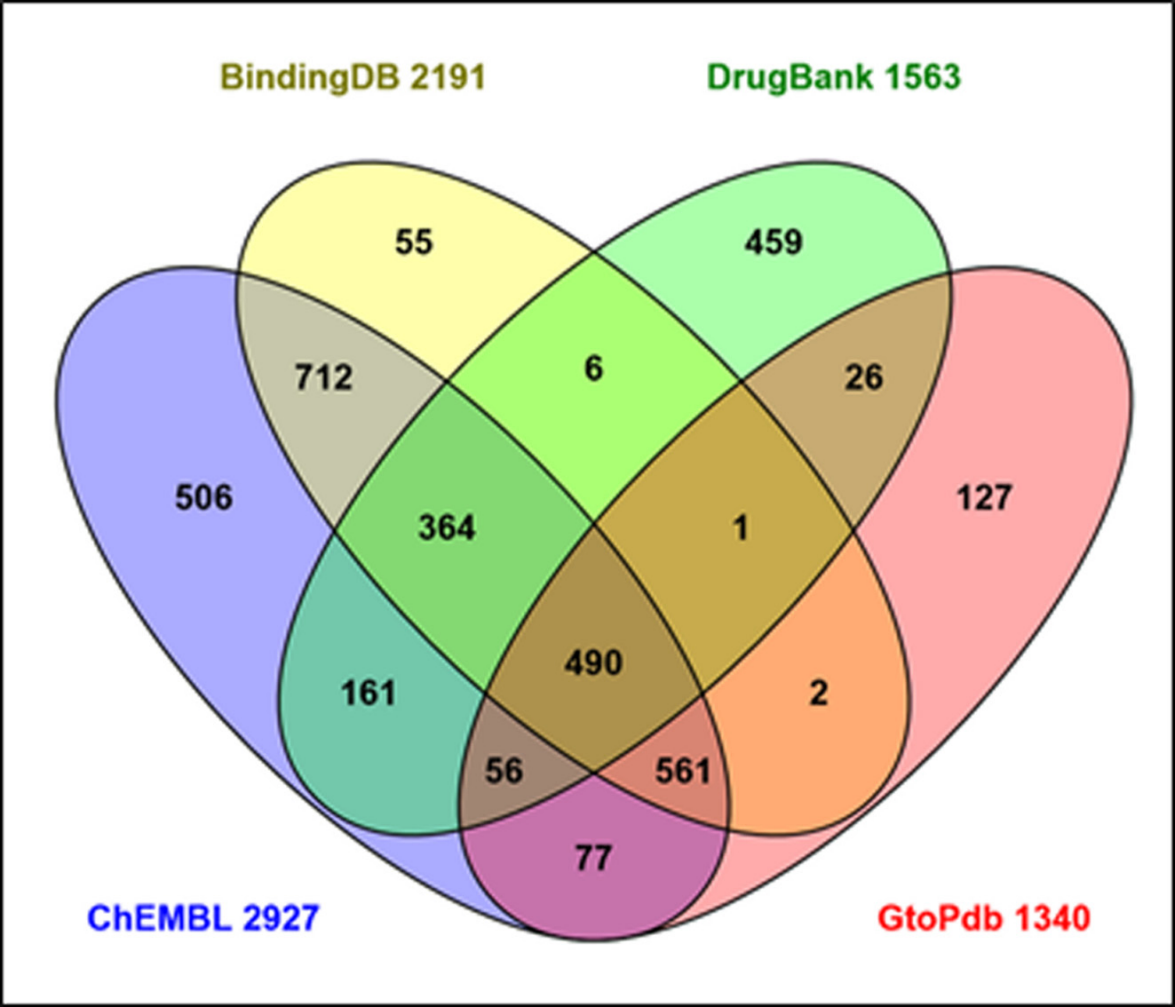
Intersects and differentials for human Swiss-Prot ID cross-referenced source databases that curate chemistry-to-protein mappings. Data were generated *via* the UniProtKB interface and the diagram prepared using the Venny tool (http://bioinfogp.cnb.csic.es/tools/venny/). The union of all four sets is 3603, based on the Swiss-Prot ID cross-references from UniProtKB release 2015_07.

Our content of targets with quantitative ligand interactions constitutes a *de facto* druggable genome. The difference is that our 1228 target interactions are supported by data rather than possible chemical modulation being merely inferred *via* transitive extrapolation. So where might the upper limit be that we could expect to achieve with our stringent but successful curation model? One source of data to address this question is Swiss-Prot where key sources of curated chemistry-to-protein mappings, including our own, can be compared. The result is shown in Figure 8.

The union of the four sources covers 18% of the human proteome. However, caveats (many of which are detailed in a 2013 database comparison study (21)) indicate this figure should be considered a maximum count. The proportion that would match our own criteria for quantitative mapping is difficult to estimate, since the chemistry-to-protein curation strategies and source selections for each database diverge considerably. This is manifest in the relatively high unique content of 1147 (31% of the union). While they converge as a four-way intersect for only 490 proteins (13.5% of the union), concordance between at least two sources (i.e. the non-unique proportion) expands to 2456. Notwithstanding, a capture goal of 2000–2500 data-supported targets for GtoPdb seems plausible. This number is particularly relevant to the ‘Illuminating the Druggable Genome (IDG) Program’ recently launched by the National Institutes of Health (NIH) (https://commonfund.nih.gov/idg/index). This is designed to expand our understanding (and drug targeting possibilities) of thinly annotated GPCRs, NHRs, ion channels and kinases. This specifically applies to ‘orphans’ within those classes hitherto without good chemical probes for function. The fit with our objective is clear. However, it remains to be seen, when and what data will surface that could be of use for curatorial expansion of the druggable genome within GtoPdb.

We plan to add enhanced query building functionality to the website allowing users to paste in lists of identifiers to retrieve targets and ligands, to choose their selection of output fields and build customised downloads. This will be accompanied by development of new browsing options and alternative entrance portals presenting a subset of the data but linked to the main database and designed for specific target audiences. One such example would combine information on targets, diseases and drugs relevant to immunology with tools to access pharmacological data. Furthermore, we are exploring options for providing access to our data in Resource Description Framework (RDF) format, which can be readily integrated in semantic web projects such as OpenPHACTS (38).

## DATA ACCESS

GtoPdb is available online at http://www.guidetopharmacology.org under the Open Data Commons Open Database License (ODbL) (http://opendatacommons.org/licenses/odbl/), and its contents are licensed under the Creative Commons Attribution-ShareAlike 3.0 Unported license (http://creativecommons.org/licenses/by-sa/3.0/). Information on linking to our pages is provided at http://www.guidetopharmacology.org/linking.jsp. We aim for three database public releases per year: the statistics quoted in this paper are from release 2015.2 (i.e. August 2015). The number of entries we deprecate between releases is low, but in rare instances an entry revision could result in a dead link in a past release. Our downloadable files include all target lists, NC-IUPHAR nomenclature, synonyms, genetic information, protein identifiers and other database accessions. Ligand downloads include isomeric SMILES and InChI strings that can be used to generate structure-data (SD) files. We can be contacted regarding other file formats or some of the custom data slices specified in recent slide presentations (enquiries@guidetopharmacology.org). Users can also download our UniProtKB and HGNC cross-links. A simple PubChem query (‘IUPHAR/BPS Guide to PHARMACOLOGY’[SourceName]) will retrieve our entire CID content (those wishing to source our local database links for these should use the corresponding SID query). The PubChem records should be synced within approximately two weeks of our release date but note it may take a little longer for all pre-computed relationships to be fully indexed.

To further facilitate distribution, we have developed an application program interface (API) in the form of REST web services to provide computational access to the data. This uses JavaScript Object Notation (JSON) as a lightweight data-interchange format that is simple for humans to read and write as well as for machines to parse and generate. JSON can be readily integrated into other websites using JavaScript. In the past, we have made an SQL dump file for download. This remains available but in response to user requests we have added a MySQL (Oracle Corporation, Redwood Shores, CA, USA) version migrated from PostgreSQL (http://www.postgresql.org/). This was created using MySQL Community Server version 5.6 on Windows, and the migration conducted with MySQL Workbench 6.2. Note that usage requires UTF-8 4-byte support using the utf8mb4 character set. We also plan to enhance our Entity Relationship Diagram for advanced users.

Since our 2014 publication, we have noted that our content has been integrated into various academic resources including CARLSBAD (39) and ChemProt 2.0 (40). In addition, we have also been informed of incorporation into some pharmaceutical company knowledgebases, such as the AstraZeneca internal Chemistry Connect system (Dr Plamen Petrov, personal communication) (41). We would ask groups (academic or commercial) interested in incorporating our data into their own resources, to contact us at the outset of their integration process so that we can assist with any technical issues that might arise on our side. The retirement of IUPHAR-DB (the precursor of GtoPdb) over two years ago (42) still produces global persistence and propagation problems. Redirects have been applied wherever possible, but users need to be circumspect if they come across secondary sources that still include IUPHAR-DB identifiers (if you notify us we can contact the parties concerned about substituting GtoPdb links).

## CITING THE RESOURCE

Please cite this article rather than previous ones; citation advice for specific target pages appears on the website. Please refer to our resource on first mention by the full correct name (IUPHAR/BPS Guide to PHARMACOLOGY) including the capitalisation. For subsequent abbreviation, please use GtoPdb and specify the release version number.

## DEDICATIONS

We dedicate this paper to the late Professor Emeritus Anthony J. Harmar (1951–2014), the founder of this resource.
